# When cardiomyopathy, cancer, and COVID‐19 collide: A case report

**DOI:** 10.1111/tbj.14182

**Published:** 2021-02-01

**Authors:** Lynn Symonds, Poorna Natarajan, Richard K. Cheng, Diana L. Lam, Mark Kilgore, Janice Kim, Kristine Calhoun, Elina Minami, Julie R. Gralow

**Affiliations:** ^1^ Division of Medical Oncology University of Washington School of Medicine Seattle WA USA; ^2^ Division of Cardiology University of Washington School of Medicine Seattle WA USA; ^3^ Department of Radiology University of Washington School of Medicine Seattle WA USA; ^4^ Department of Laboratory Medicine and Pathology University of Washington School of Medicine Seattle WA USA; ^5^ Department of Radiation Oncology University of Washington School of Medicine Seattle WA USA; ^6^ Department of Surgery University of Washington School of Medicine Seattle WA USA

**Keywords:** breast cancer, COVID‐19, cardiomyopathy, heart transplant

## Abstract

Malignancy has historically prohibited solid organ transplant; however, patients with effectively treated, favorable‐risk cancers should not necessarily be eliminated as transplant candidates. These cases require careful review by a multidisciplinary team. Here, we report the case of a woman with end‐stage heart failure undergoing heart transplant evaluation during the COVID pandemic who was found to have early‐stage, hormone receptor‐positive breast cancer. Given her favorable cancer‐related prognosis, a multidisciplinary committee recommended lumpectomy, accelerated partial breast irradiation, and adjuvant aromatase inhibitor therapy for definitive treatment to allow for consideration of orthotopic heart transplant.

## 1 CASE PRESENTATION

**FIGURE 1:**
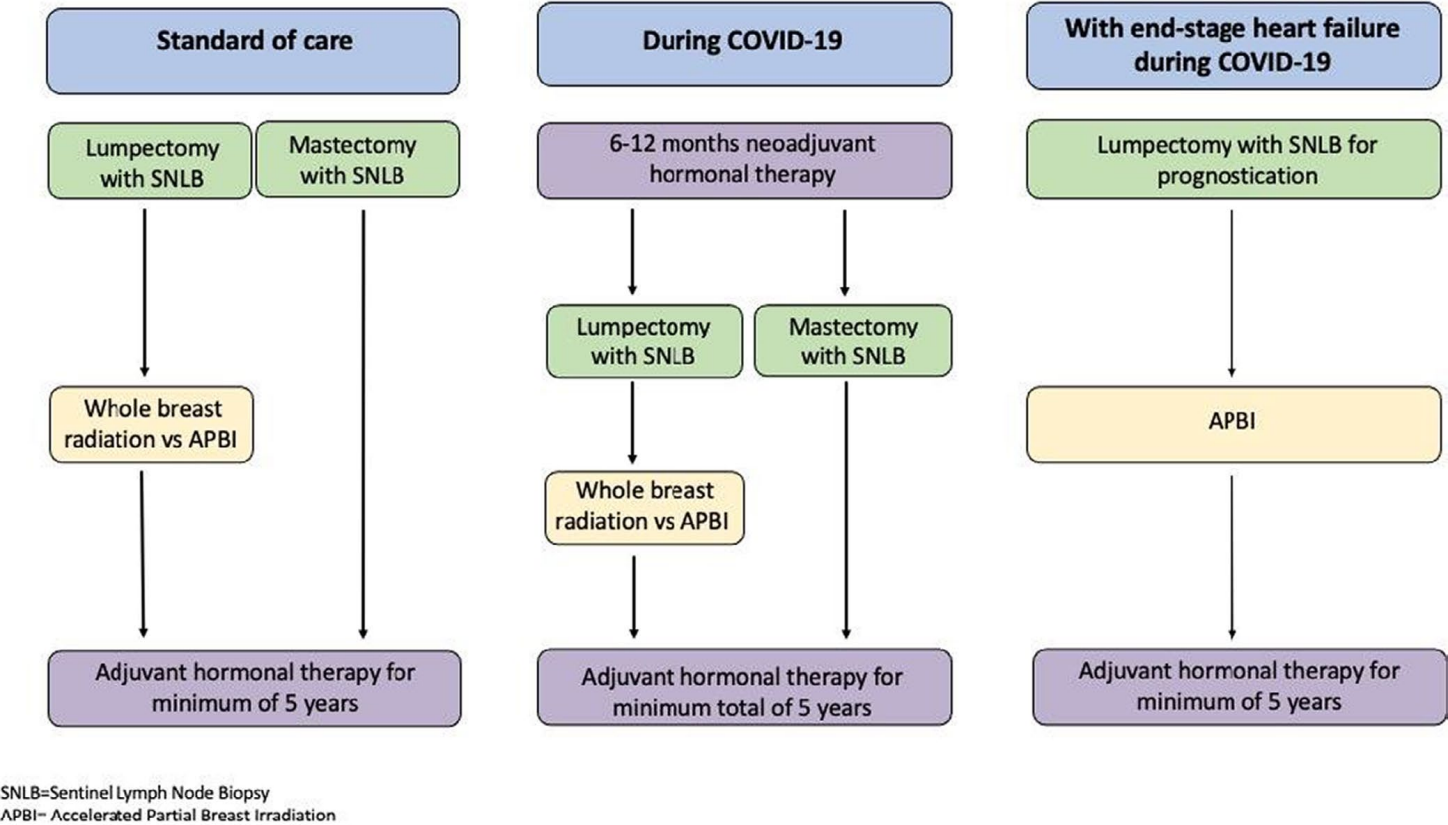
Treatment algorithms for patients with early‐stage ER/PR+, HER2‐ cancer comparing standard of care to the approach during the COVID‐19 pandemic and the proposed approach for a patient with end‐stage heart failure who presented during COVID‐19^1^

A 58‐year‐old woman with end‐stage heart failure presented in cardiogenic shock. Heart transplant evaluation uncovered a prior mammogram with unevaluated left breast architectural distortion. Inpatient breast ultrasound showed an irregular 8 × 6 × 5 mm mass. Biopsy confirmed invasive ductal carcinoma (IDC), grade 2, estrogen and progesterone receptor positive (>95%), HER2 2+ negative by FISH. Her case was reviewed at a multidisciplinary tumor board including the cardiac transplant team. Discussion included transplant eligibility, cancer treatment options, prognosis, and COVID‐19 policies. The team recommended surgery followed by radiation and endocrine therapy. She underwent lumpectomy with sentinel lymph node biopsy. Pathology showed 1.8 cm of IDC, 0/3 nodes involved (pT1pN0). She then received adjuvant accelerated partial breast irradiation (APBI) and began an aromatase inhibitor. Following breast cancer treatment, her cardiac condition deteriorated. She developed sustained ventricular tachycardia (VT) resulting in loss of consciousness which required bystander CPR and defibrillator shocks. She was readmitted and her VT was felt to be due to worsening LV failure. She was therefore re‐presented to the Heart Transplant Selection Committee and approved for heart transplant listing given excellent estimated 5‐year survival for her breast cancer (Figure 1).

## 2 DISCUSSION

Pre‐existing malignancy has been a barrier to solid organ transplant; however, early‐stage malignancies can be highly curable and should not represent an absolute contraindication. This patient's early‐stage breast cancer required thoughtful discussion of cancer treatment options given her cardiac condition and implantable defibrillator. The challenges of this case were magnified during the COVID‐19 pandemic.

One challenge was the optimal type and timing of surgery. Ultimately, lumpectomy was recommended despite requiring radiation, given greater wound healing concerns with mastectomy, and to minimize anticoagulation interruptions. Omission of nodal evaluation was considered, since results were unlikely to change radiation and chemotherapy recommendations. However, a sentinel lymph node procedure was recommended to provide cancer prognostic information for transplant eligibility. During the COVID‐19 pandemic, guidelines recommended preoperative endocrine therapy for disease control in early‐stage, ER + patients to delay surgery and hospitalization.^1^ While long‐term disease control likely could have been achieved with endocrine therapy alone, this patient's greatest chance for cardiac transplant eligibility relied on being declared cancer‐free. Another consideration for oncologic surgical timing was the possibility the patient would require a left ventricular assist device (LVAD) which would mandate continuous anticoagulation and encroach on the left breast surgical site. The decision was made to perform lumpectomy prior to possible LVAD placement to avoid anticoagulation interruption and risk of LVAD thrombosis.

Adjuvant radiation following breast conservation surgery reduces local recurrence. Discussion ensued as to whether, with negative margins, radiation could be omitted given her cardiac condition. However, data do not support excluding adjuvant radiotherapy in young patients with favorable‐risk early‐stage breast cancer.^2^ Additionally, transplant eligibility relied on an aggressive attempt to maximize cure. This resulted in proceeding with radiation. Risk of cardiac vasculopathy was considered. As radiation‐related vasculopathy typically occurs after >5 years, and this patient was expected to undergo LVAD and/or transplant soon, risk versus benefit favored radiation. Given this patient's cardiac history and implantable defibrillator, APBI was recommended using American Society of Therapeutic Radiation Oncology (ASTRO) consensus guidelines.^3^ APBI was chosen to avoid scatter to the chest/heart, decrease treatment‐related toxicity, minimize risk for defibrillator resets, and minimize delays to her cardiac treatment. After successful completion of radiation to the left breast, she started adjuvant hormonal therapy with a daily oral aromatase inhibitor therapy for a planned 5‐year duration.

Based on the National Health Services (NHS) PREDICT model, her estimated 5‐year mortality from cancer is 1% with standard‐of‐care locoregional and endocrine therapy.^4^ In contrast, median survival for inotropic‐dependent heart failure without LVAD or transplant is <1 year.^5^ She was ultimately listed and underwent successful cardiac transplantation.

## 3 CONCLUSIONS

Patients with effectively treated malignancies should not be systematically eliminated as transplant candidates, but should be individually considered on a case‐by‐case basis. Pre‐existing comorbidities require a thoughtful cancer treatment approach to minimize toxicity while providing maximal survival benefit to allow for transplant eligibility. Such cases should be reviewed by a multidisciplinary team including cardiology and oncology.

## CONFLICT OF INTEREST

The following authors have conflicts of interest to disclose: JRG: Roche/Genentech (DSMC, Steering committee), Astra Zeneca (DSMC, consultant), Novartis (DSMC), Puma (advisory board), Immunomedics (DSMC), Radia (DSMC), Pfizer (advisory board), InBiomotion (advisory board), Sandoz/Hexal (Consultant), Genomic Health (advisory board). The remaining authors have no conflicts of interest.

## Data Availability

Data sharing not applicable to this article as no datasets were generated or analyzed during the current study.

## References

[1] Dietz JR , Moran MS , Isakoff SJ , et al. Recommendations for prioritization, treatment, and triage of breast cancer patients during the COVID‐19 pandemic. the COVID‐19 pandemic breast cancer consortium. Breast Cancer Res Treat. 2020;181(3):487‐497. 32333293 10.1007/s10549-020-05644-z PMC7181102

[2] Fisher B , Bryant J , Dignam JJ , et al. Tamoxifen, radiation therapy, or both for prevention of ipsilateral breast tumor recurrence after lumpectomy in women with invasive breast cancers of one centimeter or less. J Clin Oncol. 2002;20(20):4141‐4149. 12377957 10.1200/JCO.2002.11.101

[3] Correa C , Harris EE , Leonardi MC , et al. Accelerated partial breast irradiation: executive summary for the update of an ASTRO evidence‐based consensus statement. Pract Radiat Oncol. 2017;7(2):73‐79. 27866865 10.1016/j.prro.2016.09.007

[4] Wishart GC , Azzato EM , Greenberg DC , et al. PREDICT: a new UK prognostic model that predicts survival following surgery for invasive breast cancer. Breast Cancer Res. 2010;12(1):R1. 20053270 10.1186/bcr2464 PMC2880419

[5] Gorodeski EZ , Chu EC , Reese JR , Shishehbor MH , Hsich E , Starling RC . Prognosis on chronic dobutamine or milrinone infusions for stage D heart failure. Circ Heart Fail. 2009;2(4):320‐324. 19808355 10.1161/CIRCHEARTFAILURE.108.839076

